# Combined Fluid Shear Stress and Melatonin Enhances the ERK/Akt/mTOR Signal in Cilia-Less MC3T3-E1 Preosteoblast Cells

**DOI:** 10.3390/ijms19102929

**Published:** 2018-09-26

**Authors:** Chi Hyun Kim, Eui-Bae Jeung, Yeong-Min Yoo

**Affiliations:** 1 Department of Biomedical Engineering, College of Health Science, Yonsei University, Wonju, Gangwon 26493, Korea; chihyun@yonsei.ac.kr; 2 Laboratory of Veterinary Biochemistry and Molecular Biology, College of Veterinary Medicine, Chungbuk National University, Cheongju, Chungbuk 28644, Korea; ebjeung@chungbuk.ac.kr

**Keywords:** fluid shear stress, melatonin, chloral hydrate, nocodazole, MC3T3-E1 cells, primary cilia

## Abstract

We investigated whether combined fluid shear stress (FSS) and melatonin stimulated signal transduction in cilia-less MC3T3-E1 preosteoblast cells. MC3T3-E1 cells were treated with chloral hydrate or nocodazole, and mechanotransduction sensor primary cilia were removed. p-extracellular signal–regulated kinase (ERK) and p-Akt with/without melatonin increased with nocodazole treatment and decreased with chloral hydrate treatment, whereas p-ERK and p-Akt in FSS with/without melatonin increased in cilia-less groups compared to cilia groups. Furthermore, p-mammalian target of rapamycin (mTOR) with FSS-plus melatonin increased in cilia-less groups compared to only melatonin treatments in cilia groups. Expressions of Bcl-2, Cu/Zn-superoxide dismutase (SOD), and catalase proteins were higher in FSS with/without melatonin with cilia-less groups than only melatonin treatments in cilia groups. Bax protein expression was high in FSS-plus melatonin with chloral hydrate treatment. In chloral hydrate treatment with/without FSS, expressions of Cu/Zn-SOD, Mn-SOD, and catalase proteins were high compared to only-melatonin treatments. In nocodazole treatment, Mn-SOD protein expression without FSS was high, and catalase protein level with FSS was low, compared to only melatonin treatments. These data show that the combination with FSS and melatonin enhances ERK/Akt/mTOR signal in cilia-less MC3T3-E1, and the enhanced signaling in cilia-less MC3T3-E1 osteoblast cells may activate the anabolic effect for the preservation of cell structure and function.

## 1. Introduction

Primary cilium serves as a cellular sensory organelle and mediates mechanosensing or mechanotransduction in tissues including bone, cartilage, endothelium, and kidney [1,2,3,4,5]. The primary cilium has recently been highlighted as an organelle in vertebrate development and human genetic diseases associated with ciliary dysfunction or defects in cilia formation [1]. In bone cells including osteoblasts and osteocytes, the cilia that project from the cell surface and deflect from fluid flow are required for osteogenic and bone-resorptive responses to dynamic fluid flow or fluid shear stress (FSS) [2,6,7]. FSS-induced osteoblasts play an important role in both osteogenesis and osteoclastogenesis. However, its molecular mechanotransduction mechanism is still to be understood [2,6,7]. Malone et al. [2] reported that primary cilia mediate mechanosensing in bone cells by a calcium-independent mechanism, and Kwon et al. [7] showed that primary cilium-dependent mechanosensing is mediated by adenylyl cyclase 6 and cyclic adenosine monophosphate (AMP) without intracellular Ca^2+^ release in bone cells. In a further study, Delaine-Smith et al. [6] investigated how primary cilia respond to FSS and mediate flow-induced calcium deposition in osteoblasts. Saunders et al. described how MC3T3-E1 cells respond to oscillatory fluid flow with an increase in prostaglandin E2 release [8]. Wadhwa et al. demonstrated that FSS induces the transcription of cyclooxygenase-2 through the protein kinase A and protein kinase C signaling pathways [9].

Melatonin functions as a broad-spectrum antioxidant [10,11,12] and has anti-apoptotic and anti-autophagic effects [13,14,15,16,17]. Moreover, melatonin modulates osteogenic and adipogenic differentiation, in different kinds of mesenchymal stem cells, including dental pulp-derived stem cells and adipose-derived stem cells [18,19]. Recently, Kim and Yoo [20] reported that a combination of FSS and melatonin activates anabolic proteins through the p-ERK in MC3T3-E1 osteoblast cells. Moreover, melatonin has a significant effect on bone formation through the regulation of differentiation in osteoblasts and osteoclasts [21,22], indicating that melatonin may have the potential to regulate anabolic and catabolic responses in bone remodeling. However, the influence of melatonin combined with FSS in cilia-less osteoblasts has not been elucidated. In this study, we investigated whether combined FSS and melatonin stimulated signal transduction in cilia-less MC3T3-E1 osteoblast cells.

## 2. Results

**Figure 1:**
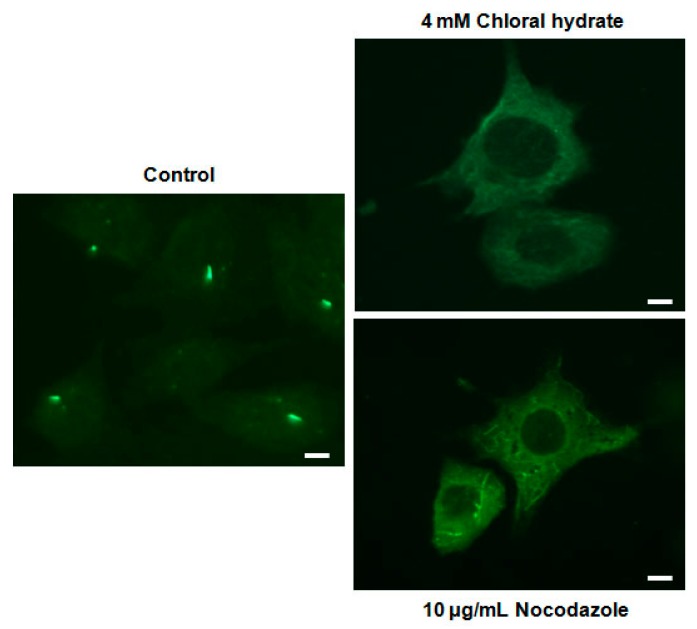
Removal of primary cilia in MC3T3-E1 cells as a sensor of mechanotransduction. MC3T3-E1 cells were treated with chloral hydrate (4 mM) for 3 days or nocodazole (10 μg/mL) for 4 h at 37 °C and 5% CO_2_. The cilia in MC3T3-E1 cells were incubated using an anti-vinculin antibody. Scale bar represents 20 μm.

**Figure 2:**
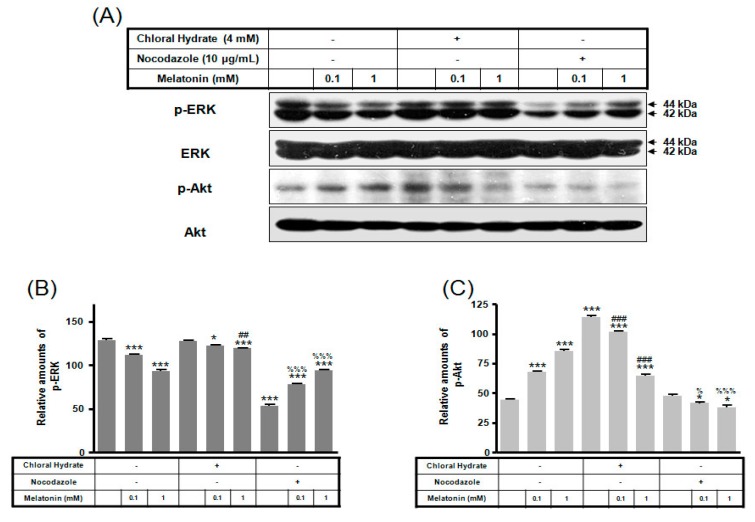
p-ERK and p-Akt expressions in MC3T3-E1 cells with/without melatonin treatment. MC3T3-E1 cells were incubated in α-MEM supplemented with 10% FBS at 37 °C with 5% CO_2_. MC3T3-E1 cells in the presence or absence of melatonin (0.1, 1 mM) were treated with chloral hydrate (4 mM) for 3 days or nocodazole (10 μg/mL) for 4 h. p-ERK and p-Akt expressions were identified by Western blots (**A**). p-ERK (**B**) and p-Akt (**C**) expressions were quantified with ImageJ analysis software. * *p* < 0.05, *** *p* < 0.001 vs. FBS alone; ^##^ *p* < 0.01, ^###^ *p* < 0.001 vs. FBS + chloral hydrate; ^%^ *p* < 0.05, ^%%%^ *p* < 0.001 vs. FBS + nocodazole.

**Figure 3:**
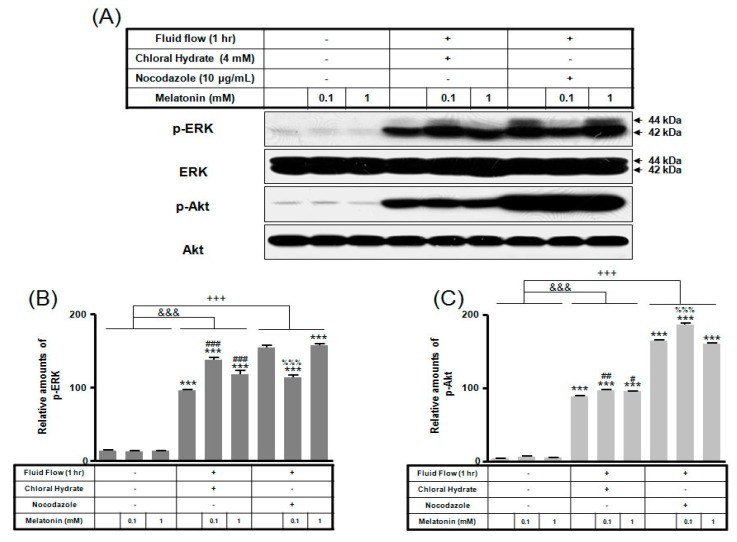
p-ERK and p-Akt expressions in MC3T3-E1 cells with/without fluid shear stress and/or melatonin treatment. MC3T3-E1 cells were incubated in α-MEM supplemented with 10% FBS at 37 °C with 5% CO_2_. Fluid flow stress experiments were performed at 1 Hz frequency and ±1 Pa maximum shear stress. MC3T3-E1 cells in the presence or absence of melatonin (0.1, 1 mM) were treated with chloral hydrate (4 mM) for 3 days or nocodazole (10 μg/mL) for 4 h. p-ERK and p-Akt expressions were identified by Western blots (**A**). p-ERK (**B**) and p-Akt (**C**) were quantified with ImageJ analysis software. *** *p* < 0.001 vs. FBS alone; ^#^ *p* < 0.05, ^##^ *p* < 0.01, ^###^ *p* < 0.001 vs. FBS + chloral hydrate; ^%%%^ *p* < 0.001 vs. FBS + nocodazole; ^&&&^ *p* < 0.001, no FSS vs. FSS + chloral hydrate; ^+++^ *p* < 0.001, FSS vs. FSS + chloral hydrate.

**Figure 4:**
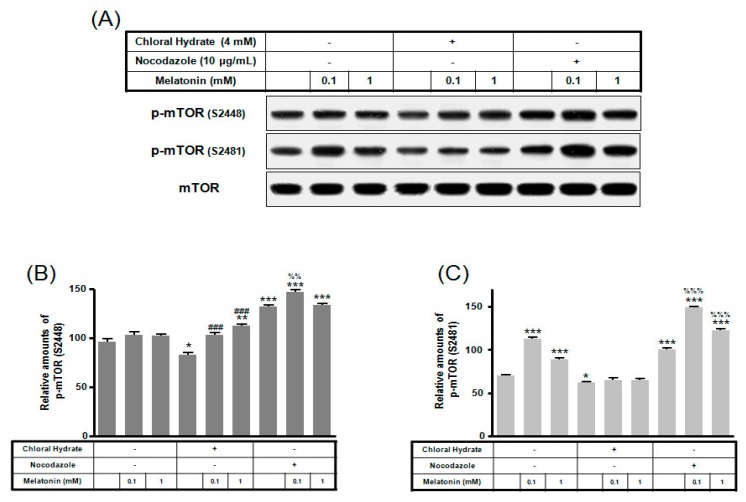
p-mTOR (Ser2448, Ser2481) expressions in MC3T3-E1 cells with/without melatonin treatment. MC3T3-E1 cells were incubated in α-MEM supplemented with 10% FBS at 37 °C with 5% CO_2_. MC3T3-E1 cells in the presence or absence of melatonin (0.1, 1 mM) were treated with chloral hydrate (4 mM) for 3 days or nocodazole (10 μg/mL) for 4 h. p-mTOR (Ser2448, Ser2481) expressions were identified by Western blots (**A**). p-mTOR (Ser2448) (**B**) and p-mTOR (Ser2481) (**C**) were quantified with ImageJ analysis software. * *p* < 0.05, ** *p* < 0.01, *** *p* < 0.001 vs. FBS alone; ^###^ *p* < 0.001 vs. FBS + chloral hydrate; ^%%^ *p* < 0.01, ^%%%^ *p* < 0.001 vs. FBS + nocodazole.

**Figure 5:**
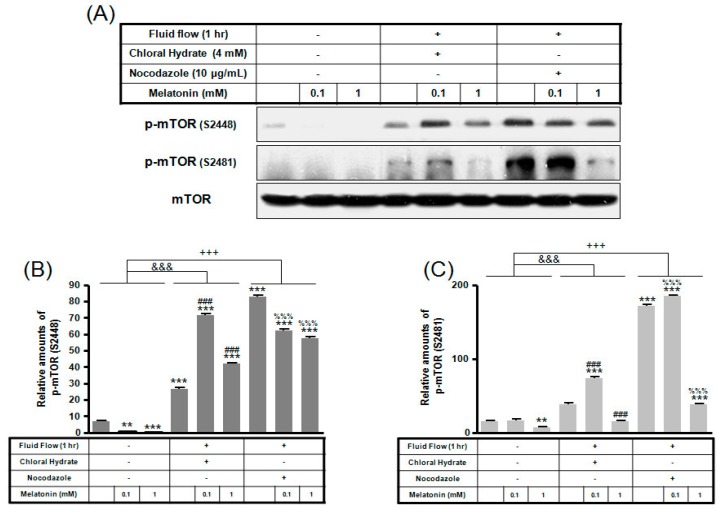
p-mTOR (Ser2448, Ser2481) expressions in MC3T3-E1 cells with/without fluid shear stress (FSS) and/or melatonin treatment. MC3T3-E1 cells were incubated in α-MEM supplemented with 10% FBS at 37 °C with 5% CO_2_. Fluid flow stress experiments were performed at 1 Hz frequency and ±1 Pa maximum shear stress. MC3T3-E1 cells in the presence or absence of melatonin (0.1, 1 mM) treated with chloral hydrate (4 mM) for 3 days or nocodazole (10 μg/mL) for 4 h. p-mTOR (Ser2448, Ser2481) expressions were identified by Western blots (**A**). p-mTOR (Ser2448) (**B**) and p-mTOR (Ser2481) (**C**) were quantified with ImageJ analysis software. ** *p* < 0.01, *** *p* < 0.001 vs. FBS alone; ^###^ *p* < 0.001 vs. FBS + chloral hydrate; ^%%%^ *p* < 0.001 vs. FBS + nocodazole; ^&&&^ *p* < 0.001, no FSS vs. FSS + chloral hydrate; ^+++^ *p* < 0.001, FSS vs. FSS + chloral hydrate.

We investigated whether the combination of FSS and melatonin stimulated signal transduction in cilia-less MC3T3-E1 preosteoblast cells. MC3T3-E1 cells were treated with chloral hydrate (4 mM) for 3 days or nocodazole (10 μg/mL) for 4 h, and then its primary cilia, as sensors of mechanotransduction, were removed (Figure 1). p-ERK and p-Akt with/without melatonin treatment (0.1, 1 mM) were increased with nocodazole treatment and decreased with chloral hydrate treatment (Figure 2), whereas p-ERK and p-Akt in FSS with/without melatonin were increased with cilia-less groups compared to cilia groups (Figure 3).

p-mTOR (Ser2481) with/without melatonin treatment was decreased in chloral hydrate treatment, and p-mTORs (Ser2448, Ser2481) were increased in nocodazole treatment (Figure 4). In FSS-plus melatonin treatments, p-mTORs (Ser2448, Ser2481) were significantly increased in cilia-less groups compared to only melatonin treatments in cilia groups (Figure 5). These data indicate that combination with FSS and melatonin enhance ERK/Akt/mTOR signal in cilia-less MC3T3-E1.

**Figure 6:**
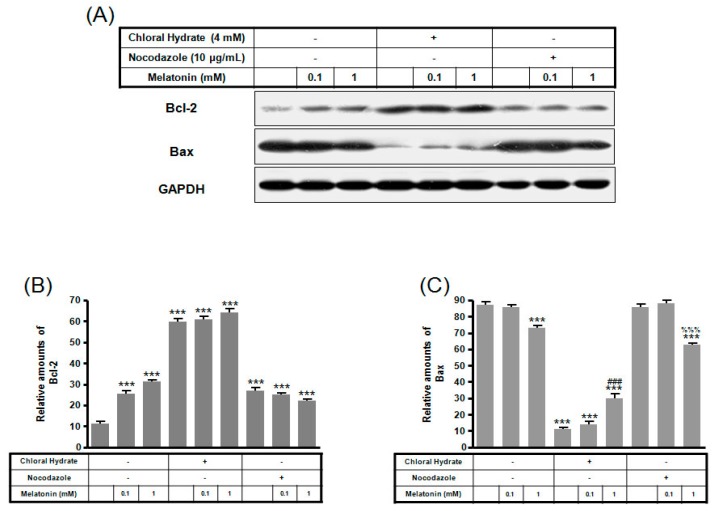
The expressions of Bcl-2 and Bax proteins in MC3T3-E1 cells with/without melatonin treatment. MC3T3-E1 cells were incubated in α-MEM supplemented with 10% FBS at 37 °C with 5% CO_2_. MC3T3-E1 cells in the presence or absence of melatonin (0.1, 1 mM) were treated with chloral hydrate (4 mM) for 3 days or nocodazole (10 μg/mL) for 4 h. Bcl-2 and Bax expressions were identified by Western blots (**A**). Bcl-2 (**B**) and Bax (**C**) were quantified with ImageJ analysis software. *** *p* < 0.001 vs. FBS alone; ^###^ *p* < 0.001 vs. FBS + chloral hydrate; ^%%%^ *p* < 0.001 vs. FBS + nocodazole.

**Figure 7:**
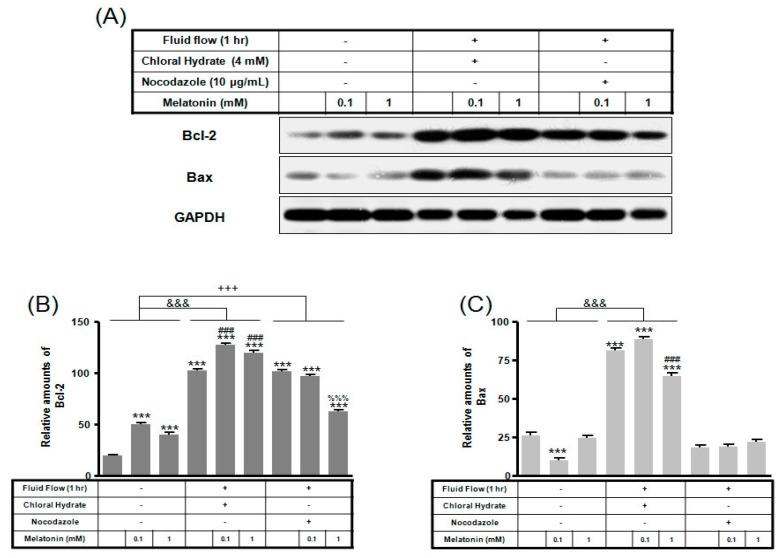
The expressions of Bcl-2 and Bax proteins in MC3T3-E1 cells with/without fluid shear stress and/or melatonin treatment. MC3T3-E1 cells were incubated in α-MEM supplemented with 10% FBS at 37 °C with 5% CO_2_. Fluid flow stress experiments were performed at 1 Hz frequency and ±1 Pa maximum shear stress. MC3T3-E1 cells in the presence or absence of melatonin (0.1, 1 mM) treated with chloral hydrate (4 mM) for 3 days or nocodazole (10 μg/mL) for 4 h. Bcl-2 and Bax expressions were identified by Western blots (**A**). Bcl-2 (**B**) and Bax (**C**) were quantified with ImageJ analysis software. *** *p* < 0.001 vs. FBS alone; ^###^ *p* < 0.001 vs. FBS + chloral hydrate; ^%%%^ *p* < 0.001 vs. FBS + nocodazole; ^&&&^ *p* < 0.001, no FSS vs. FSS + chloral hydrate; ^+++^ *p* < 0.001, FSS vs. FSS + chloral hydrate.

Expression of Bcl-2 protein with/without melatonin treatment in chloral hydrate treatment was increased, and Bax protein expression was decreased (Figure 6). In FSS-plus melatonin treatments, expressions of Bcl-2 and Bax proteins in chloral hydrate treatment were significantly increased compared to only melatonin treatments in cilia groups, whereas expression of Bcl-2 protein in nocodazole treatment was significantly increased (Figure 7).

**Figure 8:**
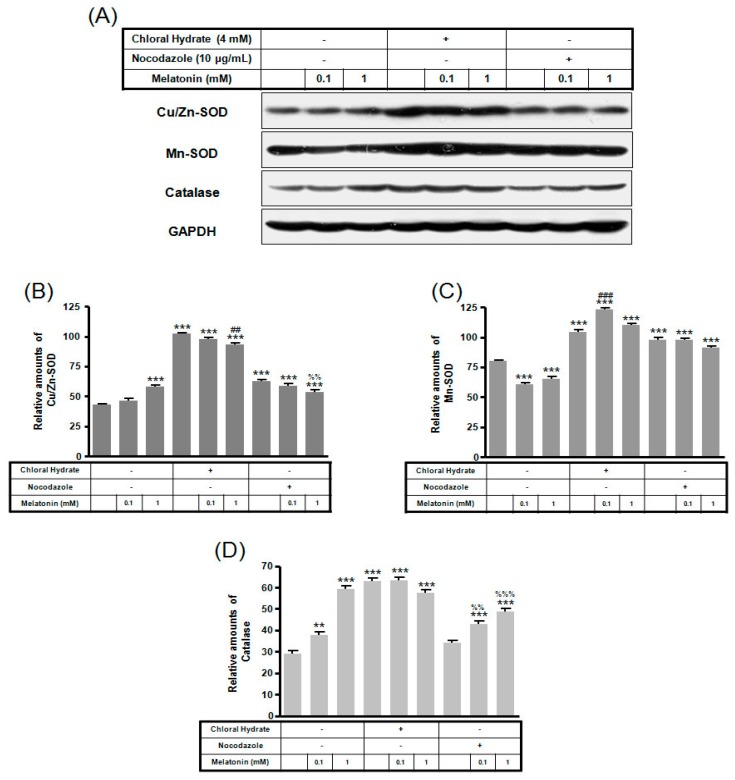
The expressions of Cu/Zn-SOD, Mn-SOD, and catalase proteins in MC3T3-E1 cells with/without melatonin treatment. MC3T3-E1 cells were incubated in α-MEM supplemented with 10% FBS at 37 °C with 5% CO_2_. MC3T3-E1 cells in the presence or absence of melatonin (0.1, 1 mM) treated with chloral hydrate (4 mM) for 3 days or nocodazole (10 μg/mL) for 4 h. Cu/Zn-SOD, Mn-SOD, and catalase proteins were identified by Western blots (**A**). Cu/Zn-SOD (**B**), Mn-SOD (**C**), and catalase proteins (**D**) were quantified with ImageJ analysis software. ** *p* < 0.01, *** *p* < 0.001 vs. FBS alone; ^##^ *p* < 0.01, ^###^ *p* < 0.001 vs. FBS +chloral hydrate; ^%%^ *p* < 0.01, ^%%%^ *p* < 0.001 vs. FBS + nocodazole.

**Figure 9:**
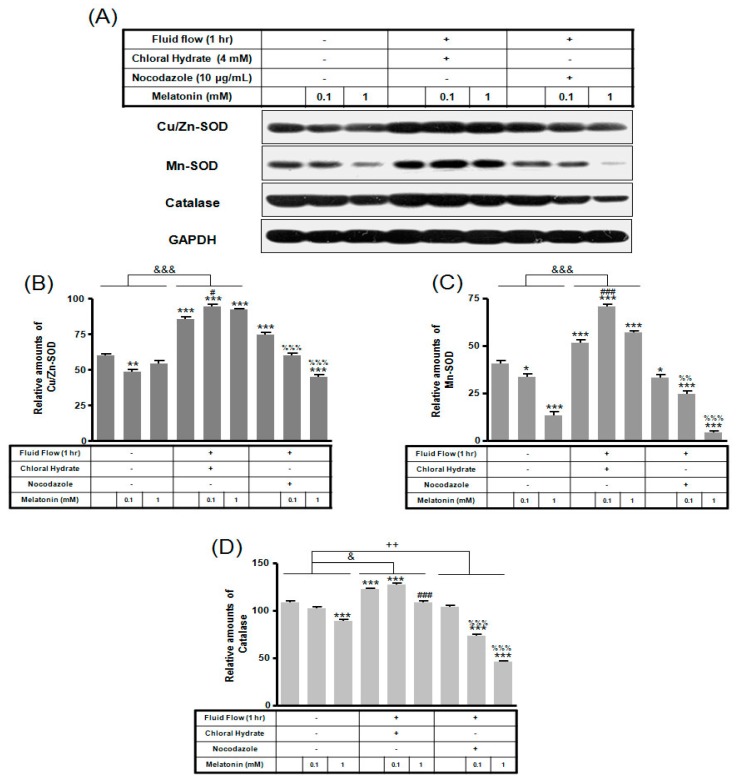
The expressions of Cu/Zn-SOD, Mn-SOD, and catalase proteins in MC3T3-E1 cells with/without fluid shear stress (FSS) and/or melatonin treatment. MC3T3-E1 cells were incubated in α-MEM supplemented with 10% FBS at 37 °C with 5% CO_2_. Fluid flow stress experiments were performed at 1 Hz frequency and ±1 Pa maximum shear stress. MC3T3-E1 cells in the presence or absence of melatonin (0.1, 1 mM) treated with chloral hydrate (4 mM) for 3 days or nocodazole (10 μg/mL) for 4 h. Cu/Zn-SOD, Mn-SOD, and catalase proteins were identified by Western blots (**A**). Cu/Zn-SOD (**B**), Mn-SOD (**C**), and catalase proteins (**D**) were quantified with ImageJ analysis software. * *p* < 0.05, ** *p* < 0.01, *** *p* < 0.001 vs. FBS alone; ^#^ *p* < 0.05, ^###^ *p* < 0.001 vs. FBS + chloral hydrate; ^%%^ *p* < 0.01, ^%%%^ *p* < 0.001 vs. FBS + nocodazole; ^&^ *p* < 0.05, ^&&&^ *p* < 0.001, no FSS vs. FSS + chloral hydrate; ^++^ *p* < 0.01, FSS vs. FSS + chloral hydrate.

In chloral hydrate treatment with/without FSS, the expressions of Cu/Zn-SOD, Mn-SOD, and catalase proteins were high compared to only melatonin treatments (Figure 8 and Figure 9). In nocodazole treatment, the expression of Mn-SOD protein without FSS was high (Figure 8), and the expression of catalase protein with FSS was low, compared to only melatonin treatment (Figure 9).

## 3. Discussion

Our recent study reported that melatonin combined with FSS activates anabolic proteins through p-ERK in MC3T3-E1 preosteoblast cells [20]. This investigation, carried out in MC3T3-E1 osteoblast cells with primary cilia under FSS and melatonin, showed that p-ERK, p-Akt, and p-mTOR (Ser 2481) expressions increased with the addition of 1 mM melatonin compared to 0.1 mM melatonin treatment. The results of the current study show that p-ERK, p-Akt, and p-mTOR in FSS with/without melatonin increased in cilia-less groups compared to cilia groups, suggesting that the enhanced signaling in cilia-less MC3T3-E1 preosteoblast cells may be activated when combined with FSS and melatonin.

In this study, primary cilia were removed with chloral hydrate or nocodazole in MC3T3-E1 cells, demonstrating that the increase of phosphorylation of ERK/Akt/mTOR in cilia-less MC3T3-E1 osteoblast cells under FSS may be activated for the preservation of cell structure and function. Delaine-Smith et al. proved that damage or removal of primary cilia with chloral hydrate inhibited fluid flow-induced mineral/calcium deposition, suggesting that primary cilia were a mechanosensor in bone cells, and highlighting their relevance in clinical treatments of bone disorders caused by dysfunctional responses to loading [6]. Jeon et al. [23] showed that osteoblastic cells with primary cilia by fluid flow stress induced an increase of COX-2 level and PGE2 release via focal adhesions and Akt phosphorylation. Malone et al. [2] suggested that primary cilia, in response to dynamic fluid flow, regulate osteopontin gene expression and MAPK phosphorylation in bone cells via tissue-specific pathways. Praetorius and Spring [24] demonstrated that chloral hydrate did not impair the Ca^2+^ mobilization machinery in MDCK cells, indicating that the primary cilium in MDCK cells functions as a Ca^2+^ sensor. Alenghat et al. [25] reported that nocodazole-treated kidney epithelial cells remove the fluid flow-induced intracellular calcium response, suggesting that disrupting the cytoskeleton in the cytoplasm may influence the function of the primary cilium to maintain its mechanotransduction response. Furthermore, nocodazole impairs tubulin polymerization in human HT-29 colon carcinoma cells, indicating that cellular interactions with the cell cytoskeleton are strongly influenced by fluid flow shear stress [26].

MAPK signaling is activated in osteoblasts that are stimulated with FSS [27,28,29,30,31,32], and Ca^2+^ change from extracellular Ca^2+^ entry or intracellular Ca^2+^ release is important for ERK activation in osteoblasts [30,33]. However, primary cilium-dependent mechanotransduction in bone cells is mediated by adenylyl cyclase 6 and cyclic AMP without intracellular Ca^2+^ release in bone cells [2,7], whereas another study showed that primary cilia under FSS mediate flow-induced calcium deposition in osteoblasts [6]. Thus, the mechanotransduction pathway on primary cilium dependence and independence may not be unique to bone cells but activates the anabolic effect under FSS [20,34]. For example, bending the primary cilium by suction with a micropipette or by increasing the flow rate of perfusate has been shown to increase extracellular Ca^2+^ in kidney cells, whereas manipulation of the apical membrane does not [35]. Praetorius and Spring also demonstrated that the flow-induced Ca^2+^ response is not inhibited by removal of the primary cilium with 4 mM chloral hydrate treatment [35].

FSS-induced activation of the phosphoinositide-3 kinase (PI3K)/Akt pathway may promote anabolic responses in osteoblasts [20,36,37]. Rangaswami et al. [38] reported that FSS-induced osteoblasts activate Akt/ERK signaling with the anabolic response of bone. Triplett et al. [39] found that FSS may regulate IGF-I-activated p-Akt and p-ERK signaling in osteoblasts. These studies demonstrate that Akt phosphorylation is required for primary, cilia-mediated, FSS-induced upregulation of osteogenic responses. In the present study, p-Akt in FSS with/without melatonin were increased with cilia-less groups compared with cilia groups in MC3T3-E1 cells (Figure 3). Similar to p-ERK increase, FSS in osteoblast activates p-Akt as an important stimulator in the cilium-dependent and cilium-independent mechanotransduction pathways.

Kim and Yoo [20] demonstrated that FSS and melatonin in combination increase the expression of anabolic proteins through the Akt/mTOR in MC3T3-E1 osteoblast cells. Lee et al. [40] provided insights into the mechanisms by which oscillatory shear stress induces osteoblast-like MG63 cells proliferation through the upregulation of PI3K/Akt/mTOR/p70S6K pathways. However, the relevance between the osteoblast-signaling pathway and anabolic proteins expression in response to mechanical stimuli is not currently known. Our present study demonstrated that FSS induces phosphorylation of mTOR in osteoblast cells with/without cilium.

In chloral hydrate treatment with/without FSS, expressions of Bcl-2, Bax, Cu/Zn-SOD, Mn-SOD, and catalase proteins were high compared to only melatonin treatments (Figure 7, Figure 8 and Figure 9). In nocodazole treatment, expression of Mn-SOD protein without FSS was high (Figure 8), and expression of catalase protein with FSS was low, compared to only melatonin treatments (Figure 9). The effects of FSS increasing antioxidant proteins including Bcl-2, Cu/Zn-SOD, Mn-SOD, and catalase in osteoblast or osteocyte cells are not well known [20,41]. Therefore, it is necessary to study antioxidant proteins under FSS.

In conclusion, we found that combined FSS and melatonin enhance ERK/Akt/mTOR signal in cilia-less MC3T3-E1. The increase of p-ERK/p-Akt/p-mTOR may have resulted from the total influence of combined FSS and melatonin in MC3T3-E1 osteoblast cells with and without cilia, and, especially, the enhanced signaling in cilia-less MC3T3-E1 osteoblast cells may activate the anabolic effect required for the preservation of cell structure and function.

## 4. Materials and Methods

### 4.1. Cell Culture

MC3TC-E1 osteoblast cells were purchased from ATCC (Manassas, MD, USA) and cultured in α-minimum essential medium (α-MEM; Gibco BRL, Gaithersburg, MD, USA) with 10% heat-inactivated fetal bovine serum (FBS; Gibco BRL, Gaithersburg, MD, USA) at 37 °C with 5% CO_2_.

### 4.2. Treatment of Chloral Hydrate or Nocodazole and Fluid Flow-Induced Shear Stress

Cells at a density of 1 × 10^6^ cells were placed in glass slides under sterile conditions. Fluid flow stress was produced by a syringe that was driven by an actuator at a frequency of 1 Hz and a maximum shear stress of ±1 Pa. MC3T3-E1 cells in the presence or absence of melatonin (0.1, 1 mM) with the treatment of chloral hydrate (4 mM) for 3 days or nocodazole (10 μg/mL) for 4 h were incubated at 37 °C and 5% CO_2_ for 1 h. Control cells were placed in flow chambers for 1 h with no fluid flow.

### 4.3. Immunofluorescence

Fixed cells were incubated overnight at 4 °C with a vinculin monoclonal antibody (clone 7F9, 1:330, Millipore, San Diego, CA, USA) and incubated with FITC-conjugated goat anti-mouse antibody (1:200, Millipore, San Diego, CA, USA). Fluorescence-labeled cells were visualized by fluorescence microscopy (Carl Zeiss, San Diego, CA, USA).

### 4.4. Western Blot Analysis

Cells were harvested, washed two times with ice-cold PBS and then resuspended in 20 mM Tris-HCl buffer (pH 7.4) containing 1% NP-40, 0.1 mM phenylmethylsulfonyl fluoride, 5 µg/mL aprotinin, 5 µg/mL pepstatin A, 1 µg/mL chymostatin, 5 mM Na_3_VO_4_ and 5 mM NaF. The cell lysate was centrifuged at 13,000× *g* for 20 min at 4 °C. Protein concentration was determined using the BCA assay (Sigma, St Louis, MO, USA). Proteins were separated by Tris-Glycine SDS-PAGE and then transferred to a polyvinylidene difluoride (PVDF) membrane. The membrane was incubated with antibodies, indicated as follows: p-Akt and Akt (1:1000, Cell Signaling Technology, Beverly, MA, USA); p-ERK and ERK (1:500, Santa Cruz Biotechnology, Santa Cruz, CA, USA); mTOR (1:500, Santa Cruz Biotechnology); Bax and Bcl-2 (1:500, Santa Cruz Biotechnology); catalase, Cu/Zn-SOD, Mn-SOD (1:1000, Cell Signaling Technology), and GAPDH (1:1000, Assay Designs, Ann Arbor, MI, USA). The membrane was exposed to X-ray film; protein bands were scanned and measured using ImageJ analysis software (version 1.37; Wayne Rasband, NIH, Bethesda, MD, USA), and normalized by GAPDH, an internal control.

### 4.5. Statistical Analysis

Data analysis was performed with Prism software (GraphPad Software Inc., San Diego, CA, USA). Values are presented as means ± SD and considered statistically significant when *p* < 0.05.
